# Reference Ranges for Hematological and Biochemical Profile of Martina Franca Donkeys

**DOI:** 10.3389/fvets.2020.602984

**Published:** 2020-12-18

**Authors:** Francesca Trimboli, Ippolito De Amicis, Antonio Di Loria, Carlotta Ceniti, Augusto Carluccio

**Affiliations:** ^1^Interdepartmental Services Centre of Veterinary for Human and Animal Health, Department of Health Science, University Magna Græcia of Catanzaro, Catanzaro, Italy; ^2^Department of Veterinary Medicine, University of Teramo, Teramo, Italy; ^3^Department of Veterinary Medicine and Animal Productions, University of Napoli Federico II, Naples, Italy

**Keywords:** donkey, reference ranges, hematobiochemical profile, healthy, Martina Franca breed

## Abstract

The Martina Franca donkey (MFd) is one of the largest Italian donkey breeds, considered as endangered breed. To support the conservation strategies, knowledge about the physiologic hematological parameters of MFds is needed. The aims of the study were to determine reference value for hematological and major serum parameters in a population of healthy MFds and to estimate the influence of age on these parameters. Eighty-one clinically healthy MFds (17 males and 64 females) in different ages were enrolled: group A (foals, n° 16, animals < 1 year old) group B (young animals, n° 36, from 1 to 3 years old), and group C (adult animals, n° 29, over 3 years old). Red blood cell count (RBC); hematocrit value (HCT); hemoglobin concentration (HGB); mean corpuscular volume (MCV); mean corpuscular hemoglobin (MCH); hemoglobin concentration distribution width (HDW); RBC distribution width (RDW); total white blood cell (WBC); WBC differential count for neutrophils, lymphocytes, monocytes, eosinophils and basophils, and platelets (PLT); mean platelet volume (MPV); platelet volume distribution width; and plateletcrit (PCT) were analyzed. The biochemistry panel included aspartate aminotransferase (AST), alanine aminotransferase (ALT), alkaline phosphatase (ALP), gamma glutamyl transferase (GGT), total serum protein (TP), albumin (ALB), cholesterol (CHOL), triglyceride (TGL), blood urea nitrogen (BUN), creatinine (CREA), glucose (GLU), Ca^2+^, phosphorus, Na^+^, Cl^−^, K^+^, and Mg^2+^. The effect of age on hematological parameters was investigated using one-way ANOVA test. Age of donkeys does not influence total WBC, HGB, HCT, platelet count and MPV, and PCT (*P* > 0.05). Some leukocyte populations such as eosinophils, monocytes, and basophils showed age-linked variations (*P* < 0.05). RBC count, RDW, and HDW decrease with age whereas MCV and MCH increase. Na^+^, K^+^, Cl^−^, Ca^2+^, phosphorus, ALP, GGT, CREA, GLUC, and CHOL decrease with age (*P* < 0.05), while AST and TP showed an increase with aging (*P* < 0.05). ALB reaches the lowest values in young donkeys and returns to values of foals in older animals (*P* < 0.05). Finally, a difference among groups for BUN and TGL was not found (*P* < 0.05). The results suggest how even for the MFd breed, age is a variable that affects different hematological and biochemical parameters. Compared to other donkey and horses, the MFd breed showed some differences that clinicians involved during conservation strategies need to be consider.

## Introduction

Donkeys (*Equus asinus*) were traditionally used as working animals for transport, riding, and farm activities. In Italy, at present, data from FAO list a total of eight breeds of donkeys: Amiata, Asinara, Martina Franca, Ragusano, Sardo, Romagnolo, Viterbese, and Pantelleria, of which a very small number of pure specimens exist (1, 2). In recent years, the growing use of donkey milk especially in children allergic to cow's milk proteins (2, 3) has seen an increasing number of donkey farms throughout Italy. The Martina Franca (MF) donkey breed, an ancient native breed of Apulia famous in the past for mule production, is one of the largest Italian donkey breeds. The population of MF donkeys consists of 1,239 specimens including 132 jackasses and 698 jennies approved for breeding (1). Notwithstanding the increasing population trend, MF donkeys are considered an endangered breed (1, 4, 5). Therefore, the exact knowledge about the physiological and pathological conditions of MF donkeys is indispensable in supporting the conservation strategies adopted for this breed. Although the knowledge of species-specific normal values of hematological and biochemical parameters is essential to classifying even in donkey the health or pathological status, in the past donkeys were considered similar to horses, limiting the study on donkey-specific reference ranges of biochemical and hematological parameters (4, 5). Many factors influence blood parameters: the most important of them are age, gender, physiological status, or circadian rhythms that can strongly affect plasma concentrations of melatonin and glucose (6).

In literature, little information is available about donkey hematological and biochemical parameters. Studies that involved donkeys of different breed as Pêga (7), Ragusana (4), Kirgyz (8), or Northwestern donkeys (9) highlighted breed-related differences in some blood parameters as lymphocytes and neutrophils that are higher in Ragusana (4) than in crossbred donkeys (10).

In donkeys of the MFd breed, the reference ranges for main biochemical and hematological parameters are poor and limited to a specific physiological status such as pregnancy (5) or limited to a particular period such as the neonatal one (11). In particular, age represents an important variable that should be considered for the evaluation of the hematological and biochemical reference range; indeed, it can influence the physiological and/or pathological status of the animal.

Sgorbini et al. (10) provided age-related changes in hematological and biochemical parameters of Amiata donkey foals from birth up to the second month of life.

The investigation of hematological and biochemical parameters in endangered Balkan donkey, autochthonous of the Serbian territory, revealed significant differences in some parameters (white blood cell, mid cell, and granulocyte counts and alkaline phosphatase) related to age (12).

Another factor influencing biochemical parameters is gender. Girardi et al. (7) reported a highest total protein value in females attributing them to the physiological responses of females in puerperium and lactation. More recently, de Palo et al. (13) showed that a different breeding technique in the early life affects biochemical profiles and lipid peroxidation patterns in donkey foals. Taking into account what has been said so far, the construction of a reference range must necessarily include the effect of influenced factors as age or breed, for the appropriate interpretation of serum biochemical results.

Finally, the instrument–instrument variation of analysis methods represents a significant influencing factor for the generation of a reference range; therefore, it is also important that each laboratory develops its own normal range. However, the determination of a normal range for the principal biochemistry and hematological parameters in an endangered donkey breed population such as those of Martina Franca is not easy due to the limited number of animals, which makes it difficult to assess the effects of age, but also of sex on them. Therefore, the aim of this study was to contribute to increasing the knowledge of the physiology of the Martina Franca donkey breed using high-quality methods and taking into account different age groups, proposing reference ranges for the main hematological and biochemical parameters in a general population of Martina Franca donkeys.

## Materials and Methods

### Animals

After a complete clinical exam, 81 clinically healthy Martina Franca donkeys (17 males and 64 females) from the same breeding farm located in Puglia (Italy) were enrolled. Donkeys were divided into three groups according to their age [4]: group A (foals, n° 16, animals < 1 year old), group B (young animals, n° 36, from 1 to 3 years old), and group C (adult animals, n° 29, over 3 years old). In details, group A included 16 donkeys (five males, 11 females, mean ± SD age: 5.6 ± 3.2 months) with age between 1 and 11 months. Group B included 36 animals (12 males and 24 females, mean ± SD age: 19.7 ± 7.2 months) from 12 to 36 months of age. Group C included 29 jennies from 4 to 22 years (mean ± SD age: 8.5 ± 4.0 years). Regarding reproductive status and lactation number of donkeys, 16 were pregnant and pluripara and 48 were non-pregnant. Seven out of the 48 were pluripara and the remaining ones were nullipara.

### Blood Sample Collection and Complete Blood Count Analysis

Blood samples came from leftover samples of the previous routine clinical investigation and were collected from the jugular vein, through an 18-gauge needle (Vacutest Kima srl, Arzergrande, Italy) into 9-ml blood vacuum tubes (Vacuette, Austria) containing K_3_EDTA for the hematological exam and into 10-ml blood collection tubes containing activation clot for the biochemical profile. Blood collection was taken during the spring season in the same day from 7.00 to 10.00 a.m. to avoid the possible influence of different photoperiods on the studied parameters and then promptly transported in a cooler to the veterinary laboratory at the University of Magna Græcia (Catanzaro, Italy). Hematological exam and serum separation were promptly performed, whereas the biochemical profile and serum protein electrophoresis were performed within 24 h after blood sampling. The serum was separated by centrifugation at 1,700 × g for 10 min at room temperature and was stored at +4°C until use.

The hematological parameters analyzed were red blood cell count (RBC); hematocrit value (HCT); hemoglobin concentration (HGB); mean corpuscular volume (MCV); mean corpuscular hemoglobin (MCH); hemoglobin concentration distribution width (HDW); RBC distribution width (RDW); total white blood cell (WBC); and WBC differential count for neutrophils (NEUT), lymphocytes (LYMPH), monocytes (MONO), eosinophils (EOS), and basophils (BASO) as percentage as absolute count (n° cell × 10^3^/μL). Platelets and their indices were also analyzed and included platelet count (PLT), mean platelet volume (MPV), platelet counts (PLT), PLT volume distribution width (PDW), and plateletcrit (PCT). All hematological analyses were performed with an automatic cell counter equipped with software dedicated for veterinary blood analysis (ADVIA 2120, Siemens Healthcare Diagnostic, Germany).

The biochemistry profile was performed on an automated biochemistry analyzer (Dimension EXL, Siemens Healthcare Diagnostic, Germany) using commercial reagents (Siemens Healthcare Diagnostics, Germany) for each parameter and dedicated standards (Siemens Healthcare Diagnostics, Germany) to set the analyzer. In particular, the biochemistry panel included the determination of the following parameters: aspartate aminotransferase (AST), alanine aminotransferase (ALT), alkaline phosphatase (ALP), gamma glutamyl transferase (GGT), total serum protein (TP), albumin (ALB), cholesterol (CHOL), triglyceride (TGL), blood urea nitrogen (BUN), creatinine (CREA), glucose (GLU), calcium (Ca^2+^), phosphorus (Phos), sodium (Na^+^), chloride (Cl^−^), potassium (K^+^), and magnesium (Mg^2+^).

### Statistical Analysis

Data analysis was performed using a statistical software (GraphPad InStat version 3.10, GraphPad Software Inc., San Diego, CA). Each parameter was tested for normality applying the Kolmogorov and Smirnov method. The following statistical parameters were calculated for each group of donkeys: mean, standard deviation, median, and 2.5th and 97.5th percentiles. The reference intervals were given as mean ± 1.96 SD for parameters normally distributed while non-normally distributed parameters were expressed as median and 2.5th and 97.5th percentiles (14).

One-way ANOVA test followed by Tukey–Kramer multiple-comparison posttest was used to determine the effect of age on biochemical and hematological parameters for normally distributed data, and if data did not have Gaussian distribution, one-way ANOVA on rank test followed by Dunn's multiple-comparison posttest was used. The level of statistical significance was set at *P* < 0.05.

## Results

Donkeys included in this study have a mean age of 4 years (± 4 years) and a range of age between 1 month and 22 years.

Reference ranges for hematological parameters were described in **Table 2**. Excluding lymphocytes (% and n° cell × 10^3^/μL) and MCH, all other cell populations were not normally distributed.

Age of donkeys did not influence the number of total WBC (*P* > 0.05; Table 1), but some leukocyte populations such as EOS, MONO, and BASO showed age-linked variations. EOS was the leukocyte population mainly affected by age (*P* < 0.05): it showed a strong increment over the years (group A vs. group C) as percentage as absolute value (n° cell × 10^3^/μL) (Table 1). Variations of MONO (n° cell × 10^3^/μL) and BASO (n° cell × 10^3^/μL) were less pronounced: both tend to decrease in older (group C) than in young animals (group B).

**Table 1:** Reference range of Martina Franca donkeys were calculated by 2.5th−97.5th percentiles when values were not normally distributed or by mean ± 1.96 SD (^§^) when values were normally distributed.

**Hematological parameters**	**All donkeys (*n*°= 78)**	**Group A (*n*°= 15)**	**Group B (*n*°= 36)**	**Group C (*n*°= 29)**
**White cell line**				
WBC (×10^3^ cells/μL)	9.5–22.1	12.8 (9.8–17.5)	14.4 (9.3–24.5)	12.4 (10.2–18.5)
NEUT (%)	29.4–69.3	42.9 (39.5–64.7)	44.9 (29.6–81.0)	42.3 (29.4–60.1)
LYM (%)	18.9–57.8^§^	43.6 (23.9–43.9)	39.5 (14.9–56.8)	38.5 (25.4–50.4)
MONO (%)	2.6–8.5	4.5 (3.5–6.4)	4.0 (2.6–8.7)	3.9 (2.6–7.3)
EOS (%)	2.7–19.8	7.0 (5.4–8.5)^a^	6.7 (2.3–13.8)^a^^,^^b^	13.6 (4.9–20.2)^c^
BASO (%)	0.2–0.6	0.3 (0.2–0.4)	0.40 (0.2–0.5)	0.3 (0.2–0.6)
NEUT (×10^3^ cells/μL)	3.5–13.7	5.6 (4.4–10.6)	6.5 (3.3–15.8)	5.2 (3.6–10.9)
LYM (×10^3^ cells/μL)	2.3–8.5^§^	5.7 ± 1.6	5.6 ± 1.9	4.9 ± 1.0
MONO (×10^3^ cells/μL)	0.3–1.1	0.5 (0.3–0.8)	0.6 (0.4–1.2)^a^	0.5 (0.3–1.0)^b^
EOS (×10^3^ cells/μL)	0.3–2.8	0.7 (0.3–1.1)^a^	1.0 (0.3–2.0)^a^^,^^b^	1.8 (0.7–2.9)^c^
BASO (×10^3^ cells/μL)	0.02–0.1	0.04 (0.02–0.09)	0.05 (0.02–0.10)^a^	0.03 (0.02–0.10)^b^
**Red cell line**				
RBC (×10^6^ cells/μL)	4.2–8.6	6.6 (6.1–7.0)^a^	5.6 (3.9–9.3)^a^^,^^b^	5.1 (4.4–6.7)^b^
HGB (g/dL)	7.5–13.1	10.2 (9.2–10.3)	9.6 (7.0–14.3)	10.0 (8.8–11.8)
HCT (%)	24.3–39.6	32.1 (30.8–32.9)	31.2 (22.3–46.8)	32.3 (28.6–38.2)
MCV (fL)	40.5–66.7	48.5 (43.9–50.2)^a^	56.0 (45.8–63.6)^a^^,^^b^	62.3 (57.1–67.6)^c^
MCH (pg)	13.6–21.3^§^	14.8 (13.5–15.7)^a^	17.3 (13.9–20.0)^a^^,^^b^	19.3 (17.7–20.7)^c^
MCHC (g/dL)	29.7–39.4	30.9 (29.7–31.8)	30.8 (29.7–31.7)	30.8 (29.9–31.6)
RDW (%)	15.6–20.5	18.5 (17.4–18.7)^a^	16.8 (16.2–19.4)^b^	16.3 (15.4–17.4)^c^
HDW (g/dL)	1.6–2.1	2.0 ± 0.15^a^	1.8 ± 0.2^a^^,^^b^	1.7 ± 0.1^c^
**Platelets**				
PLT (×10^3^ cells/μL)	74–469	159 (77–325.7)	157.5 (59–478)	220 (48–450)
MPV (fL)	6.0–11.4	8.9 (7.5–10.2)	7.7 (6.3–10.7)	7.5 (6.0–11.4)
PCT (%)	0.06–0.33	0.12 (0.08–0.15)	0.13 (0.05–0.40)	0.16 (0.05–0.30)
PDW (%)	18.45–73.6	58.8 (51.1–74.7)^a^	42.7 (18.2–73.6)^b^^,^^c^	47.5 (20.2–59.3)^c^

§ *mean ± 1.96 SD*.

a,b,c *Letters identify differences among groups for P-values < 0.05*.

*Donkeys were divided in three groups according to age: group A (foals, animals below 1 year of age), group B (young animals, from 1 to 3 years of age), and group C (adult animals, over 3 years old), and hematological parameters were indicated by median (2.5th−97.5th percentiles) or by mean ± 1.96 SD*.

Overall, RBC count decreased with age (*P* < 0.05) with simultaneous increases of MCV and MCH (*P* < 0.05) and decreases of RDW (*P* < 0.05) and HDW (*P* < 0.01). Age did not show any effect on HGB and HCT (*P* > 0.05). Platelet count and their associated parameters (MPV and PCT) were not influenced by age (*P* < 0.05) excepting PDW which results lower in older with respect to younger donkeys (*P* < 0.05) (Table 1).

Reference ranges of main biochemical parameters are listed in Table 2. Most of the variables were not normally distributed with exception of ALP and AST enzymes. The serum Na^+^, K^+^, and Cl^−^ electrolytes were found different among foals (group A) and young (group B) and adult donkeys (group C), showing a decrement with age (*P* < 0.05) (Table 2). Significant differences (*P* < 0.05) were observed between groups A and B for Ca^2+^ and Phos: both have higher values in foals, and in particular, Phos concentration tends to halve in older donkeys (Table 2). In older donkeys, Mg^2+^ showed a weak increase with respect to young donkeys (*P* < 0.05).

**Table 2:** Reference range of Martina Franca donkeys were calculated by 2.5th−97.5th percentiles when values were not normally distributed or by mean ± 1.96 SD (^§^) when values were normally distributed.

**Biochemical parameters**	**All donkeys (*n*°= 78)**	**Group A (*n*°= 15)**	**Group B (*n*°= 36)**	**Group C (*n*°= 29)**
**Electrolytes**				
Na^+^ (mmol/L)	128.0–152.0	145 (132.4–166.3)^a^	139 (125.4–149.4)^b^^,^^c^	136 (130.0–148.6)^c^
K^+^ (mmol/L)	4.2–5.9	5.0 (4.2–5.9)^a^	4.8 (4.3–5.9)	4.6 (4.3–5.3)^b^
Cl^−^ (mmol/L)	96.0–120.0	107.0 (98.4–122.9)^a^	103.0 (93.6–111.1)^b^	101.0 (96.0–109.3)^b^
Ca^2+^ (mg/dL)	12.6–17.0	15.7 (13.0–20.0)^a^	14.1 (12.0–15.7)^b^	14.6 (13.1–16.5)
Phos (mg/dL)	2.0–7.7	6.1 (4.2–9.5)^a^	3.5 (2.6–5.1)^b^	2.7 (1.9–4.0)^c^
Mg^2+^ (mg/dL)	1.6–2.6	2.0 (1.6–3.1)	1.9 (1.6–2.3)^a^	2.1 (1.8–2.2)^b^
**Enzymes**				
ALP (UI/L)	85.8–392.4^§^	285.9 ± 61.2^a^	216.7 ± 74.5^b^	247.6 ± 77.7
ALT (UI/L)	14.0–23.0	17.0 (15.0–21.3)	17.0 (13.9–21.1)	17.0 (14.7–24.8)
AST (UI/L)	158.9–371.1^§^	272 (194.9–370.9)	247.5 (155.6–351.6)^a^	288 (179.0–369.4)^b^
GGT (UI/L)	26.0–69.0	49 (29.7–65.8)^a^	37.5 (25.7–69.7)	32.0 (26.0–53.7)^b^
**Protein metabolism**				
TP (g/dL)	5.5–9.3^§^	6.8 ± 0.8^a^	7.3 ± 1.1^a^^,^^b^	8.0 ± 0.6^c^
ALB (g/dL)	1.5–3.8	2.6 (1.8–4.4)^a^	2.1 (1.3–2.7)^b^	2.4 (1.9–3.0)^ac^
BUN (mg/dL)	7.0–36.0	24.0 (5.1–58.5)	13.5 (4.9–25.7)	16.0 (5.0–22.3)
CREA (mg/dL)	0.1–0.7	0.40 (0.10–0.60)^a^	0.35 (0.09–0.74)^a^^,^^b^	0.2 (0.10–0.40)^c^
**Energy metabolism**				
GLUC (mg/dL)	28–107	84.0 (60.8–118.8)^a^	58.5 (27.5–88.6)^b^	59.0 (27.4–73.5)^b^^,^^c^
CHOL (mg/dL)	42–131	102.0 (72.1–158.7)^a^	61.0 (40.5–99.0)^b^	57.0 (48.4–79.9)^b^^,^^c^
TGL (mg/dL)	10–83	37 (17.3–78.0)	28 (2.0–62.5)	32.0 (8.0–85.7)

§ *mean ± 1.96 SD*.

a,b,c *Letters identify differences among groups for P-values < 0.05*.

*Donkeys were divided in three groups according to age: group A (foals, animals below 1 year of age), group B (young animals, from 1 to 3 years of age), and group C (adult animals, over 3 years of age), and biochemical parameters were indicated by median (2.5th−97.5th percentile) or by mean ± SD*.

Donkeys under 1 year of age (group A) showed the highest ALP activity (*P* < 0.05) while ALT was not influenced by age. Moreover, AST activity was higher in older (group C) than in young (group B) donkeys (*P* < 0.05), and GGT activity was lower in older animals than in foals (*P* < 0.05).

The serum TP showed an increase with aging (*P* < 0.01) while ALB reached the lowest values in young donkeys (group B) (*P* < 0.05) and returned to the values of foals (group A) in older animals (group C) (*P* < 0.05).

Finally, the difference among groups for BUN and TGL (*P* > 0.05) was not found, as opposed to CREA, GLUC, and CHOL whose concentrations drastically decrease with aging (*P* < 0.05) (Table 2).

## Discussion

Donkeys as horses belong to the Equidae family with which they share some physiological similarities but show also some species-specific differences. Some studies were carried out to establish in donkeys reference ranges of hematological and biochemical parameters, enrolling donkeys of different breeds as Pêga (7), Ragusana (4), Kirgyz (8), or Northwestern donkeys (9). The present study investigates for the first time the hematological and biochemical reference range of Martina Franca donkeys in a general population and then evaluates the eventual influence of age on these parameters.

In our study, Martina Franca donkey's total with cells, neutrophils, and lymphocytes is constant throughout their life and did not differ between the age groups considered according to those observed in crossbred donkeys (15) but not in Ragusana where an increased absolute value of lymphocytes and neutrophils in older animals was reported (4). According to other authors (15, 16), with respect to young and old animals MF foals show the lowest concentration of eosinophils. The number of eosinophils tends to increment with age probably due to the progressive exposure of animals to parasites during their life (17). We observed a slight decrease in basophil number between young and adult animals, not observed in the only other study, to our knowledge, present in literature which provides information about basophil count (15). Finally, the monocyte population remains substantially constant throughout life in disagreement with that reported for Ragusana (4) and crossbreed (15).

RBC and RBC indexes are dynamic parameters throughout the life of donkeys and specially during the neonatal period (11, 16, 18). Unlike horses (19), in the present study we observed that foals and young donkeys do not show signs of physiological anemia: the HGB concentration remains constant throughout life despite that MCV values are below the adult range while hemoglobinization (HDW) and anisocytosis (RDW) indexes are higher than adult animals. In agreement with previous reports (4, 20), we observed greater RBC counts in under 1-year-old than in oldest donkeys. Since the HGB concentration values are constant over the life, the contemporary reduction of RBC with aging explains the increment of MCH observed in older MF donkeys.

Platelet count was not influenced by age showing reference ranges comparable to reports of other studies (16, 20). The reference range of mean platelet volume (MPV), a PLT index of heterogeneity of platelet volume, overlaps with those reported in Northeastern donkeys (21). Decrement of platelet distribution width (PDW), a measurement of heterogenicity in platelet morphology (22), was recorded in MF similarly to that reported for Ragusana donkeys (4); this reduction was related with age progression but reached a value higher than the Ragusana breed probably due to physiological reasons.

About the biochemical profile, our study showed that many analytes are age-influenced, warning about the importance in these cases to establish the appropriate reference ranges. The only two studies published on MF breed were aimed to provide biochemical blood analysis in foals immediately after parturition, from 12 h to 11 days (11), or in jennies during pregnancy (5). Therefore, our biochemicals data are the first provided for a general population of MF donkeys.

According to Bature et al. (9) with the exception of Cl^−^ not influenced by age, electrolytes and in particular Na^+^, K^+^, and Cl^−^ showed a significant decrease with advancement of age (9). Conversely, in Pêga breed donkeys these analytes showed an opposite trend (7) similar to that reported only for Cl^−^ in Ragusana breed where Na^+^ and K^+^ are not influenced by age (4).

In our study, the marker of bone metabolism tends to decrease with aging as a probably natural consequence of age-related bone remodeling decline. In particular, in Kyrgyz donkeys the highest value of serum Ca^2+^ was found in foals and the increment of serum Phos concentration gradually decays with aging (8), corroborating the observations of Caldin et al. (4), Zinkl et al. (23), and Jordana et al. (24). Generally, a high serum Phos concentration is indicative of a fast bone growth as happens during the neonatal and youth periods. Then, the Phos concentration gradually decays with aging (25), probably due to decline of bone metabolism (23). Serum Mg^2+^ concentration observed was on average higher than that reported by other authors (4, 8). Although also influenced by age, the higher ALP activity observed in MF foals under 1 year of age seems to follow the evolution bone metabolism characterized by an initial rapid growth that decreases with aging (19, 26).

Transaminase enzyme (ALT, AST) and GGT activity showed an age-related trend different to reports on donkeys by other authors (4, 7, 15). In particular, ALT activity is not influenced by age according to Caldin et al. (4) and to that observed in horses (27) and in very young foals (11), but in disagreement with that reported by Girardi et al. (7) which observed the highest value in old donkeys. In our work, AST activity reaches its lowest values in young donkeys, confirming the observations of other authors (7, 15). As reported for Pêga (7), also in MF donkeys GGT activity is higher in foals than in older donkeys differently to those reported by observed in Ragusana and crossbred donkeys (4).

Total proteins, albumin, and BUN are biomarkers of protein metabolism which provide information about the nutritional status of animals. The range for TP and ALB values in the MF donkey population was slightly higher than that described in the Kyrgyz breed (27). Moreover, MF donkey's TP concentration resulted to be age-related with higher values in older donkeys, contrary to those reported by Kisadere et al. (27) but in agreement with Girardi et al. (7). The ALB concentration showed the same trend observed in Ragusana donkeys (4): presumably, the age-related ALB concentration increment is caused by a tendency to establish a dehydration status in old animals. Finally, BUN concentrations remain constant throughout the MF donkey's life with values comparable to those reported for very young MF foals (11) while CREA drastically decreases with aging probably due to physiological reduction of muscular mass in old animals. These results partially concord to those of Caldin et al. (4) which were observed as CREA as urea levels reach the lowest values in older animals.

As also described by other authors, the MF donkeys under 1 year of age showed the highest mean values of CHOL (4, 7) and GLUC (7) concentrations and a possible explanation could be in the kind of feeding of foals that primary is milk-based. The TGL concentration in MF donkeys is not influenced by age as reported by Caldin et al. (4), but the mean values in the correspondent age group were lower than those described in Ragusana donkeys (4) probably because they were affected by different diets.

The MF donkey population examined belongs to a unique farm and so makes it impossible to estimate the effects of the feeding management or environment. The lack of evaluation of the effects of sex on biochemical and hematological parameters in this study certainly represents a limit, especially for some hematological parameters such as RBC, HGB, and HCT which in other species, including humans, notoriously are higher in males than in females (17).

Moreover, biochemical parameters as glucose or creatinine increase during the first weeks of pregnancy (5), and also RBC and HCT are higher in late pregnancy than at foaling (28). Unfortunately, the number of pregnant and nonpregnant jennies was too different to correctly analyze the effects of pregnancy on biochemical and hematological parameters representing another limitation of this study.

## Conclusions

The MF donkey is a breed considered endangered, so knowledge about the normal values of hematological and biochemical parameters is essential to classifying animal as healthy or affected by a pathological condition. This knowledge plays an important role in the strategies aimed at the conservation of endangered breeds as Martina Franca donkeys and more in general in the panorama of the biodiversity of equidae. Further studies are needed to have more data including a wider population of MF donkeys to set a valid breed-based reference range.

In conclusion, the results suggest that for the Martina Franca donkey breed, age is a variable that, influencing different hematological and biochemical parameters, needs to be better considered in order to obtain a correct clinical evaluation of the animals.

## Data Availability Statement

The raw data supporting the conclusions of this article will be made available by the authors, without undue reservation.

## Ethics Statement

Ethical review and approval was not required for the animal study because all analyses were performed in leftover blood samples from routinary investigations performed for clinical purposes and for the benefit of the patient.

## Author Contributions

FT, ID, AD, and AC designed the study. FT, ID, and CC performed the experiments. FT analyzed the data. AD, FT, ID, and AD wrote the paper with input from all authors. AC supervised this work. All authors contributed to the article and approved the submitted version.

## Conflict of Interest

The authors declare that the research was conducted in the absence of any commercial or financial relationships that could be construed as a potential conflict of interest.
